# 

**Published:** 1890-10-11

**Authors:** 


### NOTICE TO CORRESPONDENTS.

All MS., Letters, Books for Review, and other matters intended for the Editor should be addressed The Editor, The Lodge, Porchester Square, London, W.

The Editor cannot undertake to return rejected M.S., even when accompanied by stamped directed envelope.

### Notice to Advertisers and Subscribers.

All Advertisements, Orders for Copies of The Hospital, and Business Communications should be addressed to The Publisher, *not to the Editor*, The Hospital, 140, Strand, W.C.

### Bound Volumes of "The Hospital."

Vol. VI., containing full page portraits of T.R.H. the Prince and Princess of Wales, and Miss Florence Nightingale, is obtainable at 4s., post free.

Vol. VII. is also for sale.

Orders will be received for Vol. VIII., 4s. post free. Cases for binding may be had of the Publisher, at 1s. 6d. each.

### To Residents, Secretaries, and Matrons.

The Editor will be glad to have the Regulations and each Report as issued by Asylums, Hospitals, Convalescent and Nursing Institutions, as well as those of other Charities, and an early copy in duplicate of all Appeals and Festival Notices, etc., addressed to *The Editor, The Lodge, Porchester Square, London, W.* Any superintendent, secretary, matron, or other chief official who regularly sends such information may be registered, on application to the Publisher, to receive free of cost a cover for binding The Hospital in half-yearly volumes.

### BURDETT'S HOSPITAL ANNUAL FOR 1890

Is now ready, and may be obtained of the Publisher, The Hospital Offices, 140, Strand, W.C., price 2s. 6d. limp boards, or 3s. 6d. cloth. All orders must be accompanied by a remittance.

### General Charities.

[This Column is devoted to an account of work of charitable institutions other than Medical Charities. The Editor will be glad to receive reports or news of work at 140, Strand. *Philanthropy* should be plainly written on the envelope.]

The committee of the **ROYAL HUMANE SOCIETY** announced on Saturday awards to over 100 persons for gallantry in saving life.

Miss W. M. Maxwell very kindly sends a donation through us to the "**CHILDREN'S COUNTRY HOLIDAY FUND**," and we take this opportunity of saying that we will gladly undertake to forward subscriptions or donations to any institution mentioned in this column.

Mr. Fawcett's great plea for the blind was that the way to help them to spend their last years in happiness was to treat them as much as possible like other people. "Don't confine them within the walls of institutions, don't congregate them together, but let them share the same hopes and joys with those who can see." The training institutions are splendid for the young, and future generations now look forward to seeing every blind person receive a rational education, enabling him, if need be, to gain some sort of livelihood. But how about the poor old indigent people, who know nothing, and are too old to learn anything? Their lives are very sad and dull, and many of them are just an unwelcome incubus on their families. The **ROYAL BLIND PENSION SOCIETY** decree that persons gaining their livelihood by mendicity or by playing a musical instrument in the streets or ale-houses, be ineligible for their charity, but it is the respectable poor who need help, and who find it more difficult to live than those who make an income by the display of their infirmity in the streets, and it is those who are mainly helped by the Pension Society. The next election of pensioners of this charity has been appointed for November 25th at Cannon Street Hotel, when 22 pensioners will be elected by vote, and 2 by rotation.

**THE HOMES OF HOPE**, 4, 5, and 6, Regent's Square, Gray's-Inn Road, want help very badly. These homes have been working since 1860, and were organised to help the more respectable classes of women, and to enable women expecting their first confinement to find a respectable shelter. More than seventy per cent. of those admitted to the homes turn out well. Rescue work is very uphill and perplexing, often very disheartening, but the great care seems to lie in providing the inmates with plenty of healthy employment. What strikes us as the least satisfactory point about most of these kinds of institutions is that the individuality of the women and girls is to such a great extent done away with. They come in very often from a life of far too absolute freedom to a life in the home of absolute restraint. They are in many cases not allowed letters unless they are read by the secretary or one of the matrons, their dress is of the plainest description, and the slightest attempt at ornament is forbidden. Added to this, constant religious classes and so on are held during the day. All this, no doubt, taken in moderation, is excellent and needful, but you do not change a character in a few months or even in two years. Want of self-control is the origin of all that leads a woman to need help from such institutions, and during her life there she has no opportunity of exercising self restraint in any matter, for her every word and action are under constant supervision from one source or the other. Then she is suddenly found a situation in the world, and away she goes in absolute freedom again, very likely to become one of the number who do not turn out satisfactorily. Surely then there could be some medium in all this, and a larger percentage of satisfactory results ensured if the restraint were lessened as the authorities gained knowledge of the better character of the inmate, and she were allowed to try her feet as it were, before being pitched out into the world again. We fully realise the difficulties of dealing with these cases, but we are convinced that the system is too sudden; it is the same with the religious principles; religious morals are not taught suddenly, and a woman who perhaps scarcely knows what religion is, plunged into Bible classes and prayer meetings at all times gets to regard texts as part of the daily routine, whereas the high standard of Christian morality gradually led up to would come to be something really helpful.

### Scraps and Gleanings.

Knottingley collected £20 on Hospital Saturday.

Alton Hospital Sunday collection amounted to £32.

Hampshire Eye and Ear Infirmary treated 2,315 cases last year; total expenditure, £437.

Dr. Alexander Boggs, of Paris, died this week; he was one of the leading English physicians there.

Blackpool is beginning to interest itself in the proposed hospital for which a legacy of £1,000 has been left.

Dartmouth Hospital Sunday resulted in the collection of £29, an increase of £2 on last year's collection.

Mr. A. Biddulph Martin opened the new Cottage Hospital at Sidcup on September 27th; the hospital owes its origin chiefly to Dr. Poole.

Suffolk General Hospital will not have such a large hospital collection this year. The public are suspicious after the late internal strife.

The Brittany papers say that a peasant woman of Nozay, a village near Nantes, has been safely delivered of five children, who are still alive.

The *Daily Graphic* of October 1st celebrated the commencement of the hospital season by a heart-rending picture of the dental department at Guy's.

Dr. Julia Brink has been awarded £20 by the British Medical Association to help publish her pamphlet on the nourishment of the muscular tissues.

Lowestoft Hospital held its annual meeting on the last day of September. The working expenses have been reduced, and the report is in every way satisfactory.

Heywood has an Anti-compulsory Vaccination Society, which has many adherents. Six men were lately fined 1s. each, and costs, for refusing to have their children vaccinated.

The *National Review* for October contains an article on "The Knife v. Mattei," by Dr. Herbert Snow, and one on "Homicide as a Misadventure," by H. W. Hubbard, L.R.C.P.

Pastor Hansen, of the Malling Deaf-Mute Institute, Copenhagen, who acquired much reputation by his biological studies, died suddenly on Saturday evening, in his fifty-sixth year.

The Mayor of Longton is vigorous. At the luncheon following the opening of the Cottage Hospital he left his seat and went round the table begging. He got £756, a very creditable amount.

King's College Hospital.—The Warden has received the generous contribution of £1,000 from Mr. Matthew Whiting, and the ward which had been closed for want of funds will be at once re-opened.

An invention which automatically mingles any disinfectant fluid with the cistern water when used for flushing purposes, was exhibited last week in London by the inventor, Mr. George Taylor, of Liverpool.

Two of the leading physicians of St. Louis, U.S.A., petitioned the Mayor to stay some faith-healing séances that are being held there. The doctors say the persons alleged to be cured are in fact hypnotised.

The half-yearly court of the governors of Middlesbro' Infirmary was held on October 2nd. The report was satisfactory in spite of the increased expense of the opening of the ward which has been closed for some time.

The opening of the medical session was celebrated at Sir Patrick Dunn's Hospital by the opening of new wards for fever patients. Professor Bennett delivered the opening address, and told the story of the growth of the hospital.

Hebburn is bent on having a hospital of its own, and has pointed out to prominent citizens that he should consider it a privilege to grant them a free site. The prominent citizen doesn't see it, and the hospital committee is getting impressive.

The Countess of Shaftesbury, on October 3rd, opened the new premises of the Belfast Hospital for Consumption. There was a large and fashionable assembly. Dr. and Mrs. Purdon and Miss Lewers (lady superintendent) were introduced to Lady Shaftesbury.

T. A. Goodfellow, M.B. (Lond.) M.R.C.S., L.R.C.P., appointed house surgeon to the Royal Salford Hospital, in succession to W. P. Stocks, M.R.C.S., L.S.A., resigned. R. E. Lord, M.B., B.Sc. (Lond.), M.B., Ch.B. (Vict.), M.R.C.S., appointed junior house surgeon in succession to T. A. Goodfellow.

Upwards of £1,000 has been obtained in subscriptions in Preston and the neighbourhood towards the fund for building an institute for the deaf and dumb of North and North-East Lancashire. It is a condition of the gift of £5,000 for this object by Miss Cross, of Myerscough, that £2,500 shall be raised in this way by February 1st next.

Surgeon-Major Finlay writes to explain that the class for midwives held at Aldershot has practical experience in the Hospital for Soldiers' Wives and Children, founded thirty years ago. The average number of confinements is 300 per annum, and women trained at this institution have secured the diploma of the Obstetrical Society of London.

The Burial Reform Association has completed its arrangements for the Church Congress at Hull. Sermons will be preached by the Master of the Charterhouse, Hull; and by the hon. secretary, Mr. Lawrence. A public meeting will be held, and at the Conference, the Bishop of Sodar and Man, the Bishop of Sierra Leone, and the Bishop of Bedford will take part.

The Public Sanitary Inspectors of Great Britain held their annual meeting at Carpenters' Hall last Saturday, Mr. Hugh Alexander in the chair. The first meeting of the Association for the ensuing year will be held in November, when an address will be delivered by the president, Dr. Benjamin Ward Richardson, on the "Life and Labours of Sir Edwin Chadwick as a Sanitary Reformer."

A deputation from the Newton Heath Local Board have waited upon the Amalgamation Committee of the Manchester City Council to seek their assistance in a dispute that has arisen between themselves and the committee of the Monsall Hospital. The Monsall authorities recently submitted plans of some proposed extensions at the hospital. The plans were rejected by the Board, but the extensions were proceeded with.

